# Appropriate Vestibular Stimulation in Children and Adolescents—A Prerequisite for Normal Cognitive, Motor Development and Bodily Homeostasis—A Review

**DOI:** 10.3390/children11010002

**Published:** 2023-12-19

**Authors:** Nina Božanić Urbančič, Saba Battelino, Domen Vozel

**Affiliations:** 1 Faculty of Medicine, University of Ljubljana, Vrazov trg 2, 1000 Ljubljana, Slovenia; nina.bozanic@kclj.si (N.B.U.); saba.battelino@kclj.si (S.B.); 2 Department of Otorhinolaryngology and Cervicofacial Surgery, University Medical Centre Ljubljana, Zaloška 2, 1000 Ljubljana, Slovenia

**Keywords:** epigenetic processes, pediatrics, exercise, proprioception, dizziness, vertigo, sedentary behavior, screen time

## Abstract

The structural development of the vestibular part of the inner ear is completed by birth but its central connections continue to develop until adolescence. Their development is dependent on vestibular stimulation—vestibular experience. Studies have shown that vestibular function, modulated by experience and epigenetic factors, is not solely an instrument for body position regulation, navigation, and stabilization of the head and images but also influences cognition, emotion, the autonomous nervous system and hormones. To emphasize the importance of appropriate vestibular stimulation, we present a literature review of its effect on bodily homeostasis, cognition and emotion.

## 1. Introduction

The vestibular system is one of the most essential senses, proven by the fact that it is the most ancient sensory system in vertebrates. In early vertebrates, the inner ear only provided balance information [1].

Moreover, studies have demonstrated that the auditory portion of the inner ear, which, in *Homo sapiens*, is of utmost importance for everyday functioning, has evolved out of its vestibular part [2].

The vestibular part of the inner ear consists of semicircular canals and otolith organs that enable the navigation of the head and body in different environments and stabilize a picture on the retina during head movements. This is accomplished by the vestibulo-ocular, vestibulo-cervical and vestibulo-spinal reflex [3]. Structural inner ear development is completed by birth, but the central parts of the vestibular system continue to develop until adolescence.

Vestibular information, in contrast to other sensory information, is spread to different parts of the central nervous system, forming the vestibular system-associated network. There is evidence that vestibular information also influences emotions [4,5,6], cognition [6,7,8,9,10,11], the endocrine system [12], the autonomous system [13,14] and memory and cognition [15].

We have highlighted the known facts about prenatal and postnatal vestibular development to improve our understanding of the importance of the vestibular system for everyday functioning. We have summarized the known facts about the interplay of the vestibular system network and its influence on various hormones, cognition and emotions. Since neuronal circuits of all sensory systems are immature at birth, their development depends on sensory stimulation during critical periods [16]. Studies have shown that vestibular impaired subjects also have cognitive deficits. The question is whether the lack of vestibular stimulation could have similar consequences [17].

We wanted to emphasize that a sedentary lifestyle, becoming common among children and adolescents in the contemporary world [18], is not only harmful for the metabolic aspect of their health [19] but could interfere with critical periods and have an influence on motor development and central vestibular connections and impact cognitive functions. Moreover, it could cause changes in genetic material that could be passed from generation to generation [20,21,22].

It is essential to be aware of vestibular system functions, its connections and phases of development to be able to use vestibular tests at a certain age appropriately and to educate pediatricians, parents and caregivers about the importance of vestibular stimulation in childhood and adolescence for the normal development of the vestibular system central connections and other systems that rely on it.

## 2. Inner Ear Prenatal Development

The first phase of human inner ear development begins before the third gestational week with the formation of surface ectoderm thickening on both sides of the embryonic hindbrain [3,23], which then invaginate to form otic placodes [3,23]. Meanwhile, the sensory system starts developing, with the epithelia emerging from the ectoderm in the cristae and maculae [1,3,23,24,25].

**Figure 1:**
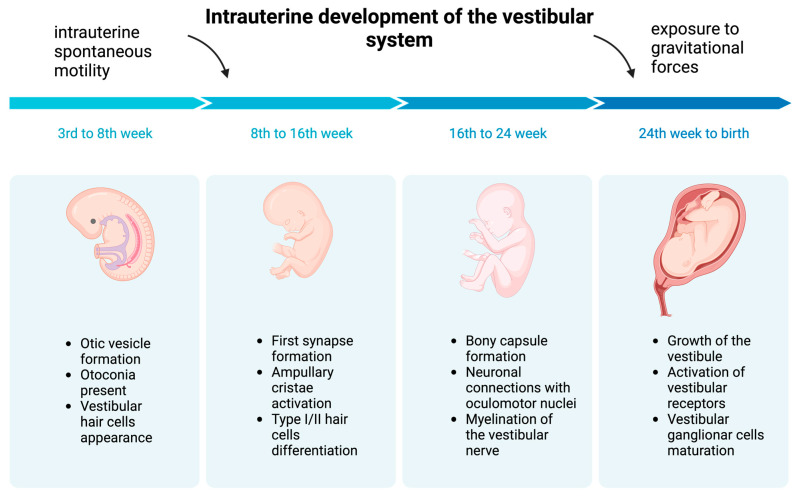
Intrauterine development of the vestibular system. Created with BioRender.com.

During the fourth gestational week, the otic placodes become surrounded by proliferating embryonic mesoderm and start creating otic pits. They start detaching from the surface ectoderm and form circle-like structures called otic vesicles [3,23] (Figure 1). During and after otic vesicle formation, cells can take on a neural, sensory or non-sensory cell fate [26]. Primary vestibular and auditory neurons are formed from the ventromedial part of the vesicles and migrate away to the future vestibular (Scarpa’s) and auditory (spiral) ganglion situated between the ear and hindbrain [27].

Local molecular events, together with a global interaction between the epithelium of the otocyst and the surrounding mesoderm, finally convert the simple otic vesicle into a three-dimensionally complicated series of interconnected sacs and tubes housing five vestibular sensory patches (i.e., three semicircular canals, utricle and saccule) and the cochlea [24].

By the seventh week of gestation, some otoconia are already present in the utricle [23] (Figure 1).

Evidence shows that the semicircular canals start receiving balance information in utero, which is vital for postnatal motor development [28]. Vestibular hair cells appear in the seventh gestational week, and the maturing crista ampullaris becomes active in the 8–9th week of fetal life [3,23].

Intrauterine spontaneous motility is observed in all vertebrates. It appears at eight weeks of gestation in humans [29]. Those automatic movements do not result from spinal reflex maturity [30]. Primitive spontaneous activity may allow cranial and spinal motor neurons to regulate muscle contractions.

At the 9–10th gestational week, the first synapse formation can be observed, with type I and type II hair cell differentiation occurring between the 11th and 13th gestational weeks [23] (Figure 1).

The bony capsule surrounding the membranous labyrinth forms rapidly from the embryonic mesoderm between the 19th and 23rd gestational weeks [23]. Around the 24th week, the development of the inner ear is complete. Consequently, the vestibule was thought to reach its adult-like form and size by the 24th week of gestation. However, recent findings suggest that the internal aperture of the vestibular aqueduct comes to its final size only after birth [23]. Vestibular receptors become active by the 32nd week of gestation (Moro reflex [31,32] can be elicited), so the vestibular afferents are mature and functional in the early stages of human development [3,23,27,33,34] (Figure 1). Vestibular ganglion cells reach maturity around the time of birth [23,35] (Figure 1).

Exposure to gravity gradually increases during the nine months within the uterus (Figure 1). Until the 21st–22nd gestational weeks, amniotic fluid prevents the gravitational forces on the body, and the fetus is in conditions similar to neutral floating in space. After the 26th week of gestation, the influence of immersion in fluid declines as the fetus starts occupying more of the intrauterine space [36]. Exposure to gravitational forces therefore intensifies, enabling the fetus to detect the vertical influences of gravity and motion [30]. This is important for adaptation and training to transition to a total gravity environment after birth; it also probably facilitates orientation during delivery. In premature-born children, the vestibulo-spinal tracts are immature, and reduced myelination is a possible long-term side effect of prematurity [37].

Preterm babies are also exposed to a dramatically altered gravity experience, without the opportunity to transition in a measured, step-by-step way.

Figure 1 presents some of the developmental periods of the vestibular system before birth.

## 3. Factors Influencing Embryonic and Postnatal Inner Ear Development

**Figure 2:**
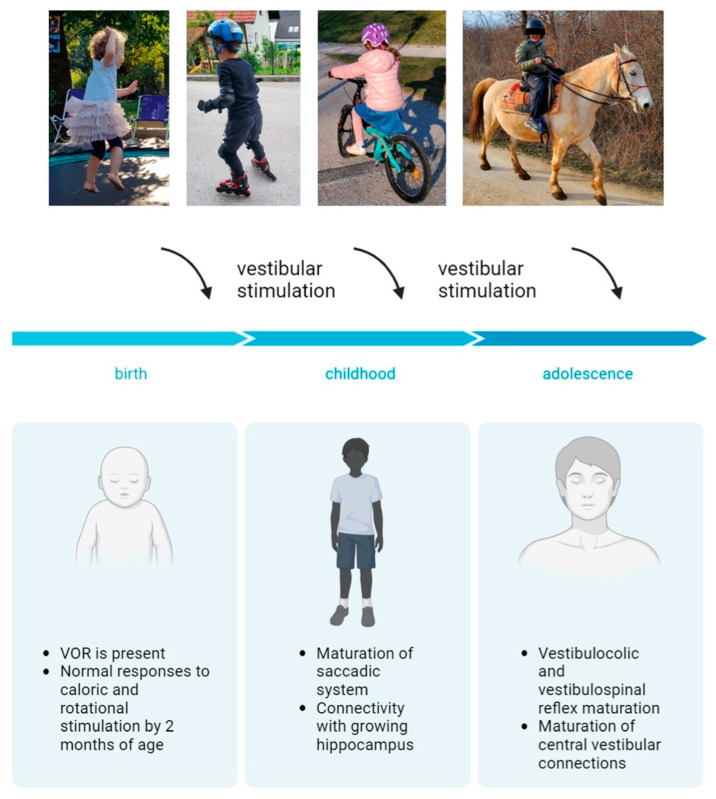
Postnatal development of the vestibular system. Created with BioRender.com.

The development of all parts of the neuronal system depends on genetics, epigenetics, experience and the external environment [38], which is essential, knowing that the impact of sensory experience on nervous system development is strongest during specific phases, called critical periods [16]. Studies have shown that vestibular-impaired subjects also have cognitive deficits. The question is whether the lack of vestibular stimulation could have similar consequences [17]. During early childhood, locomotor skills and motor development are gained through crawling, walking, playing and other experiences [30,39] (Figure 2).

Reducing gross motor activity could result in delayed achievement of motor milestones and impaired postural motor control [30]. Studies have shown the major consequences of vestibular impairment on cognition, emotion, hormonal status, etc. What is worrying is that vestibular depletion could lead to similar results.

Unfortunately, there is a global trend of increasing levels of child obesity [40] connected to sedentary behavior, primarily due to excessive screen time exposure [18]. Among the 2- to 19-year-old population of the United States, the prevalence of obesity in the years 2017 to 2020 was 19.7% [41]. A U.S. study found that children spend approximately 55% of their waking hours in non-physically active states [18]. The prevalence of sedentary behavior among European adolescents is very high (76.8%) and has not changed in the last twenty years [42]. The most alarming thing is the potential negative impact on body-weight gain, metabolic profile and diabetes. The consequence of a lack of physical activity is the loss of muscle tissue and its function, which can lead to a reduced number of vestibular synapses [43].

Consequently, motor and central vestibular system development and cognitive and emotional development could be hampered [44,45]. Unfortunately, navigation in the virtual world does not activate the same sensory or motor systems and, consequently, does not lead to appropriate vestibular stimulation [46] needed for proper vestibular development, making the next generation of gaming a solution for reducing sedentary behavior [47] but not having a positive impact on vestibular stimulation and its associated network development. Animal models have shown the necessity of active movement for normal neuronal signaling [48].

The issue of insufficient physical activity in the population has been recognized in the last twenty years [19], and global trends and campaigns aiming to introduce more physical activity to the everyday lives of children and adolescents have been initiated [19,49]. They aim to prevent the obesity and metabolic consequences of a sedentary lifestyle. The World Health Organisation recommends that children and adolescents aged 5–17 should be exposed to at least 60 min of moderate- to vigorous-intensity physical activity daily [50]. Such activities include brisk walking, jogging, uphill cycling, aerobic dance, etc.

A Singapore study found that about 17% of the population does not meet the minimum requirements for physical activity, while around half of the population spends a significant time being sedentary [51]. It has been found that children whose caregivers were more physically active enjoyed less screen time, had higher activity levels and spent less sedentary time [52].

Parents and caregivers should be educated in the need and instructed how to promote physical activity among themselves and in children and to limit screen time. Such activities, beneficial for the development of children, include playing on playground equipment, on swings that allow linear and rotary swinging, sliding on slides, jumping on trampolines, cushions, a bed, dance or movement activities, skating, swimming, etc. (Figure 2).

On the other hand, there is a tremendous genetic influence on vestibular system development and function [53]. The molecular mechanisms influencing the development and function of sensory and non-sensory components of the vestibular system are highly complex and coordinated. They involve signals originating from several tissues, including the hindbrain, neural crest, notochord, periotic mesenchyme and otic epithelium [3].

The genes involved in the early development of the inner ear have been identified, and they are found to play specific and sometimes multiple roles in the development of the inner ear [3,54]. Those are the genes involved in the specification of different cell types, the labyrinth’s orientation, endolymphal production and maintenance, the otoconia’s composition, etc. [22,32]. For example, the absence of the proteins that are the product of the genes that regulate inner ear fluid homeostasis can cause morphologic changes in the membranous labyrinth [3]. A *Notch* signaling pathway is required for the determination of hair cells and supporting cells but also for the patterning of ampulla and semicircular canals [55]. The hindbrain is an important structure for the coordination of central nervous system development and serves as an important relay station for the control of sensory and motor functions of the head [56]. The genes expressed in the hindbrain are therefore crucial in normal inner ear development. Some of these genes are *HoxA1*, *HoxA2*, *Kreisler*, *Raldh*, and *Fibroblast growth factor 3*. The consequence of their mutations is often endolymphatic duct anomalies [3]. The hindbrain genes are also required for the normal anterior-posterior positioning of the inner ear [56]. For the development of the vestibular part of the inner ear, the genes *Fgfr-2*, *GATA-3*, and *Eya1* are activated during otic vesicle formation and are expressed in the otic epithelium [3,57]. In contrast to non-mammalian vertebrates, which can produce hair cells throughout life and recover from hearing and balance deficits, embryonic production of hair cells in mammals declines sharply during intrauterine development, so deficits from hair cell losses during childhood, adolescence and adulthood are permanent [58].

Epigenetics is one way of modifying inner ear development [59]. Epigenetics studies the phenotypic changes that result from modified genetic expression rather than the changes in the genetic code itself [20,22]. They demonstrate that early life experiences, lifestyle and environmental factors can influence gene expression [20]. Consequently, motor inactivity during vestibular development can lead to epigenetic modifications [20,21,22] that may be passed on from parent cell to daughter cell or from generation to generation [60] and can influence embryonic and postnatal development [59].

## 4. Central Vestibular Development

The role of the vestibular nuclei is not only to receive and encode vestibular signals from semicircular canals and otolith organs but also a place where multisensory information from different origins merge. This information includes head, body and eye motor information, proprioceptive information and visual information [46].

Vestibular, motor and neurological development after birth can be assessed by eliciting primitive reflexes and postural reactions that reflect the integrity of the brainstem and spinal cord [31,32,61,62]. Their persistence beyond a particular age indicates delayed maturation or impaired nervous system function, and the asymmetry of the elicited reflexes suggests a central or peripheral nervous system disorder [32]. Since they reflect timely motor and neuronal development, which are tightly connected to vestibular development, they also reflect the maturity of the vestibular system. These reflexes are the Moro reflex, the tonic neck reflex, the head righting reflex, the parachute reaction and the doll’s eye response [31,32]. Postural responses represent complex motor responses to a plurality of afferences, such as the joints, the tendons, the muscles, the skin, the inner organs, the eye and the ear. The response at each chronological age is different and expresses the central nervous system maturation stage. Examination of postural reflexes has been proposed as a screening test for postural abnormalities [31,63] but can also be considered to be a vestibular function test.

**Figure 3:**
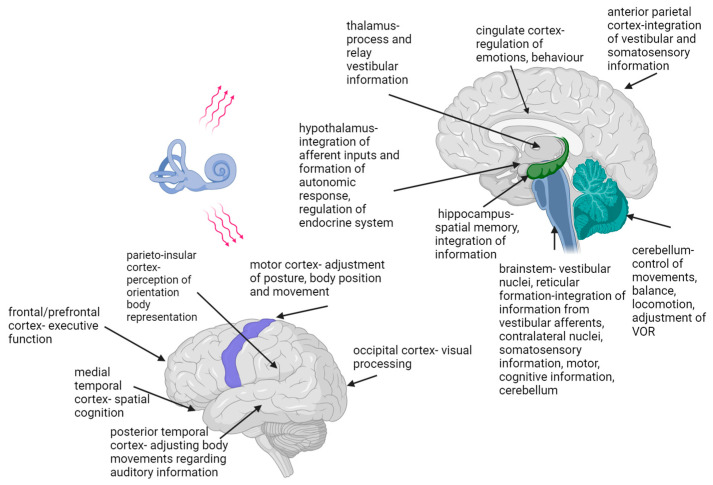
Vestibular system-associated network. The figure shows central nervous system areas known to relay information from vestibular centers [4,9,12,15,69,70,71,72,73,74]. Created with BioRender.com.

The central connections of the vestibular system and different brain areas continue to develop until adolescence. As for the other sensory systems, the thalamus is vital in central vestibular integration and is not only a relay station; the information coming from the vestibular system is dispersed to more than ten different nuclei [64] (Figure 3). Moreover, multiple projections from the vestibular nuclei to thalamic nuclei have been described, and their integration with other sensory systems has been shown [65]. The thalamus and cortex develop from different parts of the embryonic forebrain [66]. To innervate each other, their axonal projections have to grow across a complex and vast cellular terrain [67]. Corticofugal and thalamocortical projections develop synchronously at very early stages, when the distances are minimal [68]. Thalamocortical and corticothalamic projections are plastic and can reshape after alterations in sensory input or various lesions [67,68]. Understanding the mechanisms underlying their development and remodeling is vital to understanding the establishment and plasticity of cortical representations [66].

The hippocampus increases in volume during the first 6–7 years of life, with a peak at about the age of 10–11. The connectivity progresses differently in hippocampal sub-regions, reaching adult levels at 11 years of age [75]. The hippocampus is a highly plastic structure, and adults with bilateral vestibular lesions have hippocampal atrophy [76] and cognitive impairments linked to spatial and non-spatial tasks (Figure 3).

To conclude, the vestibular function continues to mature from birth onwards and reaches adult-like values at age fifteen [23]. Vestibular responses are gradually modulated by central inhibitory influences, cerebellar control and central vestibular adaptation [23]. The fine-tuning of postural adjustments is also not completed after the first walking experience. It still takes about 18 years to establish the adult capacity to modulate the temporal and quantitative parameters of postural adjustments [30].

## 5. Development of the Vestibular Reflexes

The vestibular reflexes enable gaze and posture stability during everyday activities. Mature neuronal connections, peripheral organs and vestibular nuclei are essential for their function [46]. Neuronal connections between the inner ear and oculomotor nuclei in the brainstem occur between the 12th and 24th week. Myelination of the vestibular nerve begins around the 20th week. It is the first cranial nerve to complete myelination [30]. The vestibular nuclear complex is functional at 21 weeks of gestation. The vestibulo-ocular reflex (VOR) is present at birth. Infants demonstrate inaccurate saccades, frequently requiring more than one to reach the target [32]. Maturation of the saccadic system is expected to be completed by two years of age [32]. Children have a higher gain of the VOR as a response to sinusoidal rotation than adults and, due to immature visual–vestibular interaction, also a poorer suppression of the VOR response. Most normal children have vestibular responses to caloric and rotational stimuli by two months of age. Consequently, an absence of the VOR at ten months of age is considered abnormal [77]. As a consequence of the maturation of visual pathways, the time constant approaches adult values by the age of 2 months. [32].

Compared to the VOR pathway, the vestibulo-spinal pathways are more complex because of the number of muscles and joints that are controlled, the variety of activity patterns and the biomechanical constraints, which vary considerably throughout development [1]. Vestibulo-spinal and vestibulo-colic reflexes mature gradually until adolescence [23]. The central vestibular pathways mature until 11 to 15 years of age [8]. Consequently, differences between children’s and adults’ VEMP (vestibular-evoked myogenic potential) responses can be observed [78,79], suggesting maturational effects from preschool through adolescence. It is not surprising that vestibular stimulation is crucial in that developmental period. Due to suppressed vestibular stimulation (for various possible reasons), various difficulties can appear, such as delayed acquisition of motor skills, problems with cognition, reading (further compromised language skills in hearing loss patients), altered spatial representations, etc. [8,71].

Studies have indicated that the developing nervous system cannot compensate for a vestibular deficit during the early phase of ontogeny [80] (Figure 2). Achievements during the postnatal development of the vestibular system are presented in Figure 2.

## 6. Vestibular Screening—A Necessary Tool for Timely Identification of Children with an Underdeveloped Vestibular System

Universal hearing screening has enabled timely and successful hearing rehabilitation of hard-of-hearing children, contributing significantly to normal child development, socialization and education.

Vestibular screening would enable early detection of vestibular impairment in a child, leading to appropriate vestibular rehabilitation.

The idea of vestibular screening goes back to 1974, when Isabelle Rapin published her observations when performing vestibular tests on hard-of-hearing children and concluded that vestibular assessment should not be limited only to at-risk populations [81].

The screening method should be chosen based on the child’s age at the time of the screening and the expected level of vestibular system development. The method should be applicable for routine use in pediatric outpatient clinics [82]. Some authors use the test of the vestibulo-spinal reflex (Romberg test—the ability to maintain erect posture with the eyes closed, standing on one foot, walking) as a screening method. Since the vestibulo-ocular reflex matures earlier, we should consider applying one of the tests for VOR.

Unfortunately, we are still at the same stage as we were 50 years ago, thinking about vestibular screening only for selected groups of patients (hard of hearing, patients with congenital cytomegalovirus infection, etc.).

The brain builds representations of verticality based on vestibular, somatosensory, proprioceptive and visual information. Knowing how vital the vestibular system and its central connections are for the normal development and function of balance and navigation, cognition, endocrine, vegetative system, etc., we should consider universal vestibular screening in children. Early detection of vestibular dysfunction would enable adequate and timely rehabilitation, normal postural control acquisition, gaze stabilization and prevention of secondary sequelae involving multiple systems.

## 7. The Vestibular System-Associated Network

The vestibular system is the only sensory system whose information is not directed to a single and specific brain area [83] (Figure 3). Vestibular information is spread from the vestibular nuclei to different parts of the central nervous system, forming a vestibular system-associated network [9,69,83]. Studies have proven that this network significantly impacts the function of various organs and systems, and that there are complex interactions between central vestibular fields and cognitive, oculomotor and emotional circuits [84,85]. The connections between the vestibular nuclei and frontal, prefrontal, parietal, cingulate, occipital, posterior and medial temporal cortex, insula and retroinsular cortex, thalamus, hippocampus, cerebellum, the Sylvian fissure and the brainstem have been described [14,69,70,71] (Figure 3). The vestibular network information and cognitive functions are integrated at the cortical level and within the hippocampal and limbic systems [4,6,10]. Not all of the functions of the above-mentioned cortical areas and their possible interactions and pathways are known [71]. Studies assessing vestibular-associated networks use electrical, caloric, visual or sound stimulation to induce the vestibular response [86,87,88,89,90]. The current testing methods lack the possibility of functional brain imaging during head and body movement.

On the basis of an experiment using caloric stimulation, Batchold suggested that vestibular stimulation leads precisely to enhanced functioning of the contralateral cerebral structures, the parahippocampal gyrus and the temporal lobe [89]. As Wilkinson et al. showed, electrical vestibular stimulation positively affects the speed of visual memory recollection [86]. Recent studies have indicated that non-invasive electrical stimulation causes simultaneous activation of semicircular canals and otolith systems and can improve neuronal plasticity in the central pathways [88].

Much has been learned from studies on unilaterally and bilaterally vestibular-impaired subjects. The most straightforward are gaze and fixation problems associated with vestibular dysfunction, which can cause decreased visual acuity and consequent reading problems [91]. Some studies have demonstrated that humans with vestibular disorders show a range of cognitive deficits that are not just the consequence of spatial information deficits, such as spatial navigation, spatial learning and spatial memory, but also of problems with establishing a mental representation of the world, which could be connected to impaired numerical skills in vestibular-impaired subjects [10,70,71]. These cognitive deficits also include non-spatial functions such as object recognition memory, self-motion perception and bodily self-awareness [70,92]. Other cognitive deficits related to vestibular dysfunction include the impairment of short-term memory and concentration and impaired numerical skills, attention and cognitive processing ability [10,11]. In his 2015 review, Lopez argued that vestibular information impacts the perception of the body on different levels: from the perception of touch, pain and body dimensions to the feeling of owning a body, the feeling of being placed within one’s body and the visuospatial perception of the body [93].

The visual interpretation of body posture is used to anticipate emotions and intentions and is also essential in social interactions [17].

The influence of vestibular information on emotions has been known since ancient times. Hanging beds, providing low-frequency vestibular stimulation, were used to induce sleep and relieve pain. Similarly, spinning chairs, with their high-frequency stimulation, have been used to treat mental illnesses, such as manic episodes [3,4]. In relation to the influence on emotions, vestibular dysfunction can not only induce or cause a worsening of anxiety, depression and other affective disorders, but there is evidence that affective conditions may also influence the central vestibular systems [85,94,95,96]. This is not surprising, knowing the effects of vestibular impairment on everyday functioning, daily activities, social interactions and the connections of the vestibular system with the limbic system (Figure 3). There is also robust evidence in the literature of a close functional interplay between vestibular and anxiety systems [97].

Moreover, vestibular–autonomic networks have also been described, which explain the influence of body position on autonomic regulation of blood pressure and respiratory activity [13,14,98]. Interestingly, the receptors of adrenal gland hormones, thyroid hormones, sex hormones and insulin have been identified in inner ear structures, the vestibular nerve and the vestibular nuclei [12]. Electrical activation of the vestibular system alters sympathetic system activity throughout the body, with the renal nerve being the most sensitive to vestibular inputs [13]. The insular cortex has been recognized as a cortical site for vestibular and autonomic integration and modulation [99,100].

Regarding the influence of sex hormones, studies have confirmed the influence of female sex hormones on susceptibility to peripheral and central vestibular disorders [101,102]. A study by Serra et al. found altered glucose and insulin levels in patients with peripheral vestibular disorders [103]. Congenital hypothyroidism, among other organs, also has a negative effect on inner ear development [104]. A connection between the inner ear and thyroid cells has also been found concerning exposure to gravity, which modulated the thyroid cells in a study by Grimm [105]. The positive experience that clinicians dealing with acutely vestibular-impaired patients have had with corticosteroid therapy is one of the signs of vestibule–adrenal interactions. Corticosteroids are found to accelerate compensation after vestibular loss in guinea pigs [106].

There are not as many studies exploring vestibular connections to hormonal balance as there are for other vestibular connections.

In conclusion, cortical vestibular representations are ubiquitous, and there are still unresolved questions regarding the function and pathways of some of the cortical areas known to be part of that network [70,71].

## 8. Conclusions

Vestibular system development starts in utero and continues to adolescence. Although it is considered to be one of the most “ancient” sensory systems, central vestibular pathways are still not completely understood. However, it is known that the vestibular system relays important information via central connections to multiple other systems essential for survival, motor, cognitive and emotional development and hormonal homeostasis. Moreover, activities that stimulate the vestibular system are critical for adequate development of these systems, particularly in childhood and adolescence. For that reason, any interference with normal vestibular development or lack of appropriate vestibular stimulation could be prevented by educating parents, caregivers and teachers about the importance of vestibular system stimulation through free play instead of prolonged exposure to screen time.

## Data Availability

The data presented in this study are available in article.
